# Comparison of the coexistence pattern of mangrove macrobenthos between natural and artificial reforestation

**DOI:** 10.1002/ece3.70069

**Published:** 2024-07-31

**Authors:** Pingping Guo, Yufeng Lin, Yifei Sheng, Xuan Gu, Yijuan Deng, Yamian Zhang, Wenqing Wang, Mao Wang

**Affiliations:** ^1^ Key Laboratory of the Ministry of Education for Coastal and Wetland Ecosystems, College of the Environment & Ecology Xiamen University Xiamen China; ^2^ Zhangjiang Estuary Mangrove Wetland Ecosystem Station, National Observation and Research Station for the Taiwan Strait Marine Ecosystem Xiamen University Zhangzhou China

**Keywords:** biodiversity, community assembly, macrobenthos, mangrove, mangrove restoration

## Abstract

The abandoned pond‐to‐mangrove restoration project provides greater advantages than tidal flats afforestation in restoring mangrove ecosystem services and will be the primary method for mangrove restoration in the future. The existing methods for abandoned pond‐to‐mangrove restoration include artificial restoration through ‘dike‐breaking, filling with imported soil and tree planting’ and natural restoration through ‘dike‐breaking and natural succession’. However, little is known about which restoration strategy (natural or artificial restoration) provides more benefits to the biodiversity of mangrove macrobethos. Given a prevailing view suggested that artificial restoration should be the preferred approach for accelerating recovery of biodiversity and vegetation structure in tropical regions, we hypothesised higher macrobenthic biodiversity and more complex community structure in artificial restoration than in natural restoration. To test this hypothesis, macrobenthic biodiversity and ecological processes were monitored in a typical abandoned pond‐to‐mangrove area of Dongzhaigang Bay, China, where artificial and natural restoration methods were used concurrently. Differences in macrobenthic biodiversity, community structure and ecological processes were compared using diversity indices, complex network analysis and null models. Similar species composition and ecological niche overlap and width among macrobenthos were observed at artificial and natural restoration sites. The biotic heterogeneity and interaction among macrobenthos were higher at the natural restoration sites than at the artificial restoration sites. Macrobenthos community assembly at natural and artificial restoration sites was both determined by deterministic processes, with environmental filtering dominating, which explained 52% and 54% of the variations in macrobenthic community structures respectively. Although our findings did not validate the research hypothesis, higher biotic heterogeneity and species interaction among macrobenthos could support natural restoration as the primary method for abandoned pond‐to‐mangrove projects, because it is a nature‐based solution for mangrove restoration.

## Graphical abstract

This study explored which method is more effective in restoring the biodiversity of mangrove macrobenthos in existing methods for abandoned pond‐to‐mangrove restoration including artificial and natural restorations. We found that similar species composition, lower biotic heterogeneity and species interaction among macrobenthos at artificial restoration sites than that at natural restoration sites. It provides an insight into natural restoration as the main method, with artificial restoration as a supplement in future actions of mangrove restoration.

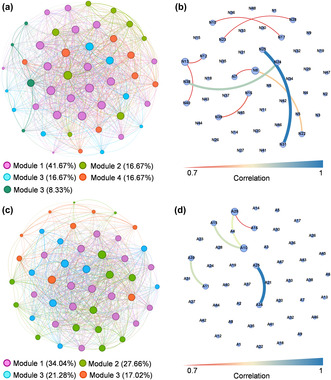

## 1 INTRODUCTION

Mangrove ecosystems are distributed in tropical, subtropical and warm temperate intertidal ecoregions (Wang & Wang, 2007). They provide crucial ecosystem services such as biodiversity maintenance, fisheries habitat, carbon sequestration, water purification and sediment accretion (Wang et al., 2021). 62% of the world's mangrove forests have been lost by deforestation, pollution and climate change in recent decades (Goldberg et al., 2020; Hagger et al., 2022). Remote sensing monitoring results indicated that more than 50% of the global mangrove loss resulted from aquaculture pond farming (Goldberg et al., 2020; Kuenzer et al., 2011). Much effort has been conducted worldwide to maintain and restore mangrove forests to slow their further decline. There are two major methods to restore mangrove forests globally: mangrove afforestation on tidal flats and abandoned pond‐to‐mangrove (Wang et al., 2021). Almost 2000 km^2^ of mangroves have been planted on global tidal flats over the past 40 years (Sasmito et al., 2023). More than 90% of increased mangrove forests during 2000–2020 were attributed to tidal flat afforestation in China. However, the afforestation of tidal flats faces various challenges, such as the depletion of suitable land for mangrove forests, increasing costs of afforestation, decreasing effectiveness of afforestation, single tree species used in afforestation and threats from pests and diseases (Lee et al., 2019; Wang et al., 2021; Zhang et al., 2021). Evidence from Southeast Asia that the biodiversity and carbon sequestration potential of mangrove restoration in abandoned ponds were significantly higher than that of tidal flat afforestation is mounting (Ahmed et al., 2017; Duncan et al., 2016; Song et al., 2023). About 8000 km^2^ of previously deforested mangrove areas globally remain biophysically suitable for restoration (Sasmito et al., 2023). Therefore, the abandoned pond‐to‐mangrove restoration should be the primary method for future mangrove restoration worldwide. However, there is a lack of research on the biodiversity of the abandoned pond‐to‐mangrove restoration.

The existing methods for abandoned pond‐to‐mangrove restoration include artificial restoration through ‘dike‐breaking, filling with imported soil and tree planting’ and natural restoration through ‘dike‐breaking and natural succession’ (Wang et al., 2021; Zhang et al., 2021). Current restoration projects of abandoned ponds for mangroves have mainly focused on the plant community structure, diversity and vegetation cover during the process of abandoned pond‐to‐mangrove restoration (Aslan et al., 2021; Oh et al., 2017; Xiong et al., 2021). However, restoring the diversity of macrobenthos is also crucial for the stability of mangrove ecosystem structure and function, as macrobenthos participate in many important ecological processes (Chen & Ye, 2011). Macrobenthos are highly regional, weakly migratory and sensitive to changes in environmental conditions and can serve as effective indicators of successful mangrove restoration (Cannicci et al., 2021; Chen, Gu, Lee, et al., 2021). Few studies compared the effectiveness of different methods in abandoned pond‐to‐mangrove restoration (Di Nitto et al., 2014). Although a recent study has compared differences in biodiversity between natural and artificial restoration sites of abandoned ponds to mangroves, little is known regarding differences in macrobenthic community structure, assembly processes and key drivers.

The metacommunity concept is a key theory for comprehensively understanding community composition and ecological processes (Lamy et al., 2021). A metacommunity is a collection of native communities that may interact through species dispersal and replacement (Leibold et al., 2004). The constant flushing of tidal waters promotes the rapid transport and exchange of nutrients and benthic propagules, connecting localised communities in the ecosystem to form a typical metacommunity (Gu et al., 2023; Lee et al., 2014). The formation and stability of macrobenthic metacommunities are influenced by local selection processes (e.g. ecological niche differentiation and environmental filtering) and spatial processes (e.g. dispersal or colonisation; Leibold et al., 2004; Thompson et al., 2020). Resolving the deterministic and stochastic nature of these processes by combining their structures and mechanisms is a new way to study metacommunities (Stegen et al., 2012). The interaction patterns and mechanisms of metacommunities are influenced by deterministic and stochastic processes and can be assessed using co‐occurrence network analyses. Deterministic processes refer to both biotic and abiotic factors that affect the presence and abundance of species and are related to ecological selection (Mark, 2010). Stochastic processes refer to environmental disturbances, probabilistic dispersal and random life and death events experienced by the community, rather than adaptation outcomes determined by the environment (Chase & Myers, 2011; Vellend et al., 2015). Deterministic and stochastic processes have been studied in various ecosystems, including tropical rainforests (Ellwood et al., 2010), temperate forests (Fang et al., 2019), grasslands (Segre et al., 2015) and lakewater ecosystems (Wang et al., 2013). However, it has been applied less in highly interconnected and open mangrove ecosystems because of the lack of field survey data, resulting in a limited understanding of the maintenance mechanisms and ecological processes of mangrove biodiversity. In addition, studying interspecies interactions with co‐occurrence networks in mangrove macrobenthic communities may help to understand the distribution patterns of species within communities, ecological niche differentiation and the evolution of communities (de Vries et al., 2018; Zhou et al., 2021). Shifts in macrobenthic network structure affect mangrove ecosystem functioning and stability (Chen, Gu, Liu, et al., 2021; Hernandez et al., 2021). However, previous studies have almost exclusively focused on single properties of macrobenthic communities and their functioning, rather than on the macrobenthic co‐occurrence networks (Zhang et al., 2021). There is also limited evidence on which or how the macrobenthic network complexity and stability respond to natural restoration and artificial restoration of mangrove ecosystems.

This study aims to explore (1) whether the diversity of mangrove macrobenthos was significantly varied between artificial and natural restoration, and (2) what ecological processes and environmental factors determined the difference. A prevailing view suggested that artificial restoration should be the preferred approach for accelerating recovery of biodiversity and vegetation structure in tropical regions (Shoo et al., 2016). Evidence indicated that artificial restoration is better than natural regeneration for the recovery of rainforest birds and plant diversity (Cardoso et al., 2022; Hariharan & Raman, 2022). In addition, owing to rapid establishment of stable ecosystems by human intervention and deterministic processes (e.g. environmental filtering and species competition), multi‐species mixed planting can considerably provide greater biodiversity and ecosystem function in the early stages of restoration (Feng et al., 2022). Whereas, the establishment of stable ecosystems by natural regeneration is a relatively slow process, which is dominated by a stochastic dynamic process of forest restoration (e.g. the colonisation of opportunistic and locally adapted species) (Chazdon & Guariguata, 2016; Crouzeilles et al., 2017). Given these evidence, we therefore hypothesised that (1) higher alpha and beta biodiversity and more complex community structure were in artificial restoration than in natural restoration, and (2) the community assembly in natural restoration was dominated by stochastic processes but by deterministic processes in artificial restoration. To test this hypothesis, we investigated the richness of mangroves, molluscs and crustaceans at the artificial and natural restoration sites in the Dongzhaigang National Nature Reserve, in which multiple mangrove tree species were planted. We compared their α‐ and β‐diversity patterns, niche overlap and interspecies interaction intensity, and identified key processes and factors determining local β‐diversity.

## 2 MATERIALS AND METHODS

### 2.1 Study area

**FIGURE 1:**
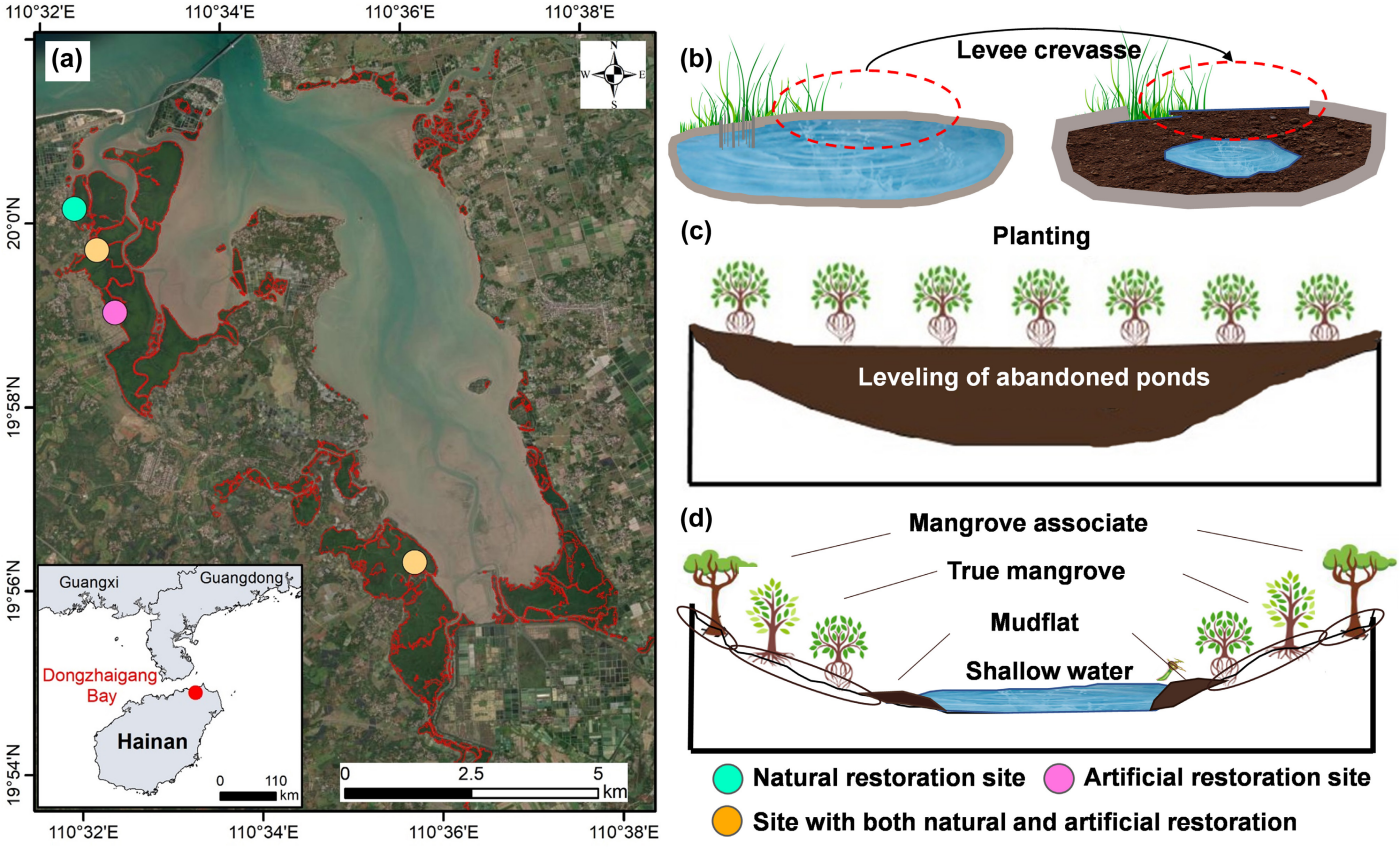
Overview of the study. (a) Location of Dongzhaigang Bay on Hainan Island, China, and the study area within the bay (coloured dots), where abandoned pond‐to‐mangrove restoration programmes were conducted from 1995 to 2018. (b) Schematic of restored hydrological conditions. (c, d) Diagram showing the two abandoned pond‐to‐mangrove restoration methods compared in this study. The artificial restoration method included hydrological restoration and planting (c), and the natural restoration method included hydrological restoration only (d).

A total of 12,923.7 ha of mangrove forests disappeared in China between 1980 and 2000, 97.6% of which was used to build aquaculture ponds. However, many aquaculture ponds were abandoned, and the total area of abandoned aquaculture ponds in China is approximately 730 km^2^ (Friess et al., 2019; Wang & Wang, 2007). Moreover, there are nearly 100 km^2^ of aquaculture ponds in mangrove conservation areas and wetland parks in China (Wang et al., 2021; Wang & Wang, 2007). An urgent need is restoring these abandoned ponds to mangrove forests. Currently, China's government has conducted some abandoned pond‐to‐mangrove restoration projects in several national natural reserves, such as Dongzhaigang Bay and Shenzhen Bay. We selected Dongzhaigang Bay as a typical case (19°51′–20°01′ N, 110°32′–110°37′ E), which is situated in the northeastern region of Hainan Island, China (Figure 1a), with a mangrove area of 1733 ha. The area of aquaculture ponds within this bay is over 1300 ha, and most are converted from natural mangrove forests (Wang et al., 2009; Zhang et al., 2021). The abandoned ponds to mangrove restoration projects with various restoration methods were conducted in Dongzhaigang Bay from 1995 to 2018 (Figure 1b–d; Zhang et al., 2021). This provides a good experimental site for comparing the structural variations in macrobenthos associated with different restoration methods. Dongzhaigang Bay has mixed semidiurnal tides, with a mean tidal range of 1.6–1.8 m (Wang et al., 2009). The dominant climate is tropical monsoon, with an annual mean rainfall of 1676 mm and a mean annual temperature of 24.8°C. The dominant tree species were similar between the natural and artificial restoration sites and were *Aegiceras corniculatum*, *Bruguiera sexangula* and *Kandelia obovata*.

### 2.2 Field surveys

Macrobenthos surveys and physicochemical measurements of sediment and seawater were conducted twice, between January 2022 and August 2023, in the wet and dry seasons. In total, 150 macrobenthos samples and 150 sediment samples were collected at artificial and natural restoration sites of abandoned pond‐to‐mangrove in Dongzhaigang Bay.

### 2.3 Macrobenthos community

Macrobenthic sampling for molluscs and crustaceans was conducted at five artificial and five natural restoration sites during low tide, with five replicate quadrats randomly established per restoration type. The molluscs were extracted from 0.25 m × 0.25 m × 0.30 m (length × width × depth) quadrats and stored in a refrigerator at <4°C. The species were identified, counted and weighed in the laboratory, and the density and biomass of the molluscs were calculated. The crustacea samples were collected using a combination of sampling and netting methods. The sampling method was consistent with that used in the mollusc surveys. In total, 150 centipede nets with 8.5 mm mesh were laid in the sampling sites before rising tides and picked up after falling tides. The crustacea species were identified, counted and weighed in a laboratory. Interstitial water salinity was measured in each macrobenthos quadrat in the in situ test (three replicates per quadrat) using a pocket digital display salinometer.

### 2.4 Environmental variables

The environmental variables were measured immediately after obtaining biological samples from each quadrat. Water temperature, pH, dissolved oxygen and turbidity were measured using a multiparameter water‐quality analyser. Relative surface elevation was measured at each site using a global navigation satellite system with real‐time kinematic positioning (G970 GNSS RTK). Surface sediments were sampled to a depth of 30 cm after collecting benthic mollusc samples from each quadrat during low tides. Soil moisture, pH, salinity, total nitrogen (TN) and total organic matter (TOM) were measured. Soil moisture was measured using the wet–dry‐specific gravity method. Soil pH and salinity were measured using pH and salinity meters, respectively, after dissolving the ground sample in deionised water for 24 h. TOC and TN were measured using an elemental analyser after sample pretreatment (Nieuwenhuize et al., 1994; Zhang et al., 2021). Plant samples were collected from 10 m × 10 m quadrats in mature mangroves and 5 m × 5 m quadrats for mangrove seedlings. The number of tree species, diameter at breast height, canopy height, and tree density were also recorded. Daily air temperature data were obtained from the National Meteorological Information Center (http://data.cma.cn/).

### 2.5 Statistical analyses

#### 2.5.1 α‐Diversity, β‐diversity and niche overlap indices

We used the Simpson index, Shannon–Wiener index and Pielou evenness to measure the α‐diversity of macrobenthos and exerted the Bray–Curtis distance metrics to measure the β‐diversity of macrobenthos. The Simpson index, Shannon–Wiener index and Pielou evenness were calculated using the Vegan package in R (version 4.2.1; www.r‐project.org). Bray–Curtis dissimilarities were evaluated based on the relative abundance data using the *Raup*–*Crick* function of the Vegan package in R 4.2.1. Non‐metric multidimensional scaling ordination based on the Bray–Curtis distance was used to investigate the differences in the β‐diversity of macrobenthos between artificial and natural restoration. We performed a randomisation/permutation procedure analysis of permutational multivariate analysis of variance to examine significant variations in macrobenthos between artificial and natural restorations (Anderson et al., 2006). These analyses were conducted using the *metaMDS* function in the Vegan R package (Oksanen et al., 2013). The niche width and niche overlap index were calculated using the SPAA package in R version 4.2.2 based on the following equation:

(1) $$O_{\mathrm{ik}}=\frac{\sum_{j=1}^{r}\left(n_{\mathrm{ij}}\times n_{\mathrm{kj}}\right)}{\sqrt{\sum_{j=1}^{r}{\left(n_{\mathrm{ij}}\right)}^{2}\sum_{j=1}^{r}{\left(n_{\mathrm{kj}}\right)}^{2}}}$$

where *n*_*ij*_ and *n*_*kj*_ are the importance values of species *i* and *k* in the *j*‐th environmental degree, respectively; *r* is the number of environmental factor gradients; and *O*_*ik*_ is the niche overlap value of species *i* and *k*.

#### 2.5.2 Co‐occurrence network construction

Correlation‐based network analysis has been widely used to infer biome interactions (Chen, Gu, Lee, et al., 2021; Hernandez et al., 2021). Specific network properties, including specific taxa, modularity and cohesion, have been used to assess network complexity and primary functions (Guo et al., 2022). We calculated Spearman correlations for species pairs to compare the co‐occurrence patterns of macrobenthos at the artificial and natural restoration sites. Based on significant correlations among the genera (Spearman's correlation coefficient *r* > .5, *p* < .05), two co‐occurrence networks for macrobenthos at the natural and artificial restoration sites were constructed using the Hmisc package in R version 4.2.1. Network visualisation was performed using Gephi software (Mo et al., 2021). The modularity, clustering coefficient, connecting lines and average degree of connection of the networks were measured to explore the assembly strategy of macrobenthos. Higher network modularisation, nodes, connecting lines and average connection degree of networks indicate higher community complexity (Grilli et al., 2017; Zhou et al., 2021).

#### 2.5.3 Deterministic and stochastic processes

We constructed Raup–Crick matrices (β_*RC*_) using the standardised Bray–Curtis community matrix to distinguish between deterministic and stochastic processes. Raup–Crick matrices indicate whether the observed dissimilarity level deviates from the null model (Chase & Myers, 2011). Raup–Crick dissimilarity values range from −1 to 1, where −1 ≤ β_*RC*_ < 0 indicates limited dissimilarity and increased similarity. Environmental filtering and homogeneous dispersal may aggregate communities with similar compositions and functional traits. When β_*RC*_ = 0, stochastic processes, such as ecological drift, may influence community assembly, leading to random structures. Furthermore, 0 < β_*RC*_ ≤ 1 indicated limited similarity and increased dissimilarity, suggesting species competition and/or dispersal limitations could contribute to segregating communities with distinct compositions and traits (Chen, Gu, Liu, et al., 2021). We calculated the proportion of β_*RC*_ values within each interval (<0, =0, or >0) to assess the relative significance of these processes. The Raup–Crick matrix was generated through 5000 random draws using the *Raup*–*Crick* function from the Vegan package (Chase & Myers, 2011) in R 4.2.2. Finally, Mantel tests were conducted to identify the relationships between mollusc communities, crustacea community dissimilarity and environmental spatial distances (Mo et al., 2018).

## 3 RESULTS

### 3.1 Macrobenthos in natural and artificial restoration exhibit similar species composition but different biotic heterogenisation

**FIGURE 2:**
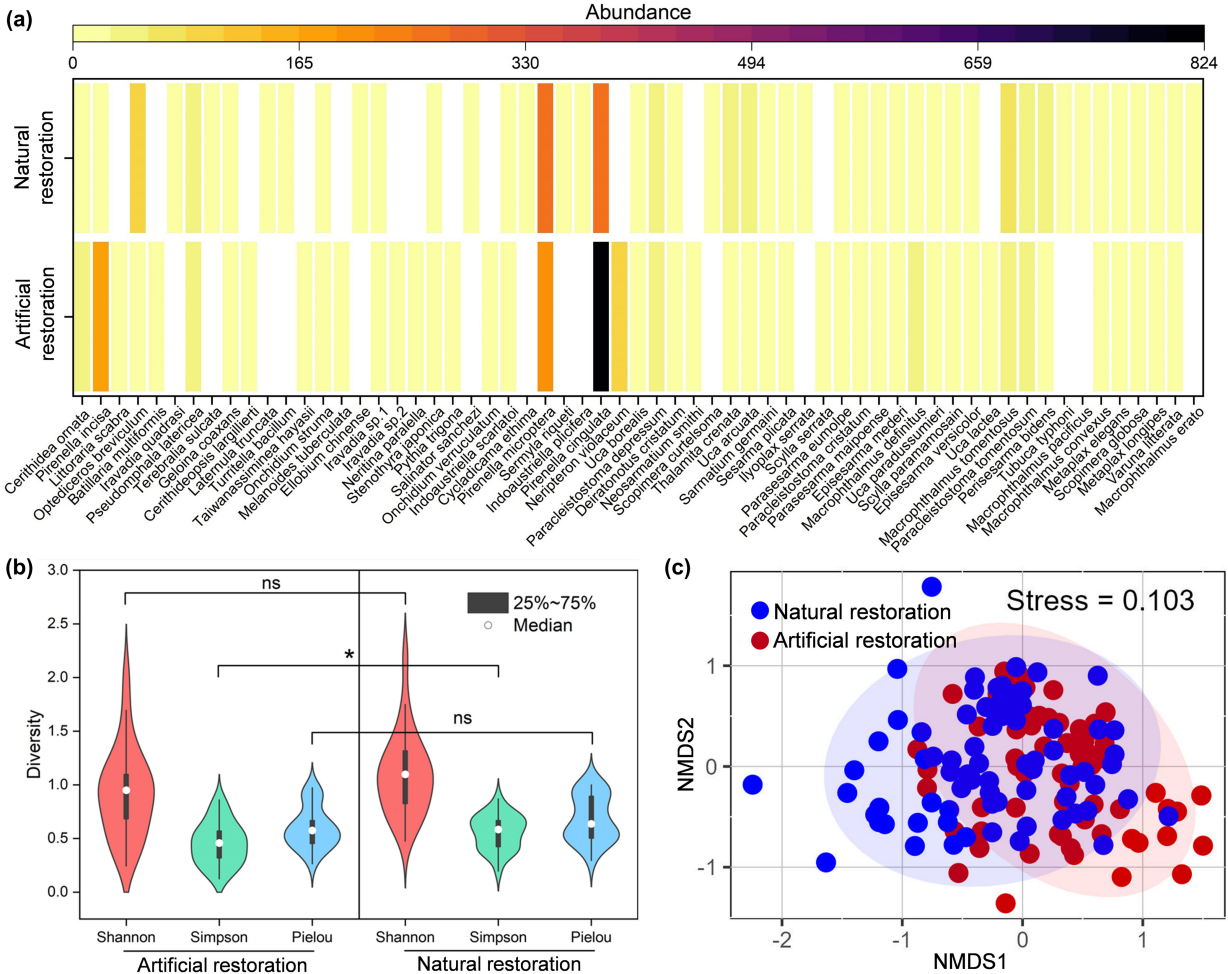
Comparison of macrobenthos species richness between artificial and natural restoration (a), observed Shannon–Wiener index, Simpson index and Pielou index of macrobenthos between artificial and natural restoration (b), and non‐metric multidimensional scaling (NMDS) ordination of macrobenthos between artificial and natural restoration (c). *, *p* < .05; ns, *p* > .05.

A total of 61 macrobenthic species (30 mollusc and 31 crustacea species) were discovered at the abandoned pond‐to‐mangrove restoration sites in Dongzhaigang Bay. In contrast, 47 macrobenthos species were discovered at artificial restoration sites and 48 at natural restoration sites (Figure 2a; Table S1). The *Pirenella microptera* and *Pirenella cingulata* were the co‐dominant species in both restoration areas. The *Potamididae* and *Ocypodidae* were the most abundant families. The Simpson index of macrobenthos communities at artificial restoration sites was significantly lower than at natural restoration sites, but the Shannon–Wiener and Pielou indices at the artificial restoration sites were similar to those at the natural restoration sites (Figure 2b). This indicated that macrobenthic communities at artificial restoration sites exhibited similar evenness and lower dominance than at natural restoration sites.

The β diversity of macrobenthos was significantly different between artificial and natural restoration (stress = 0.103, *R* = .23, *p* < .05; Figure 2c). Furthermore, the Bray–Curtis dissimilarity index of macrobenthos at the artificial restoration sites was significantly lower than that at the natural restoration sites (502 vs. 720, *p* = .04, Figure S1), indicating biological homogeneity with higher similarity of species components at the artificial restoration sites. Several species contributed to the difference in β diversity of macrobenthos between artificial and natural restoration sites, including *Pirenella microptera*, *Pirenella cingulata*, *Neripteron violaceum* and *Pirenella incisa*, with contributions of 20.06%, 7.03%, 5.68% and 5.34%, respectively (Table S2).

### 3.2 Macrobenthos in artificial restoration exhibited lower species complexity and interaction than in natural restoration

**FIGURE 3:**
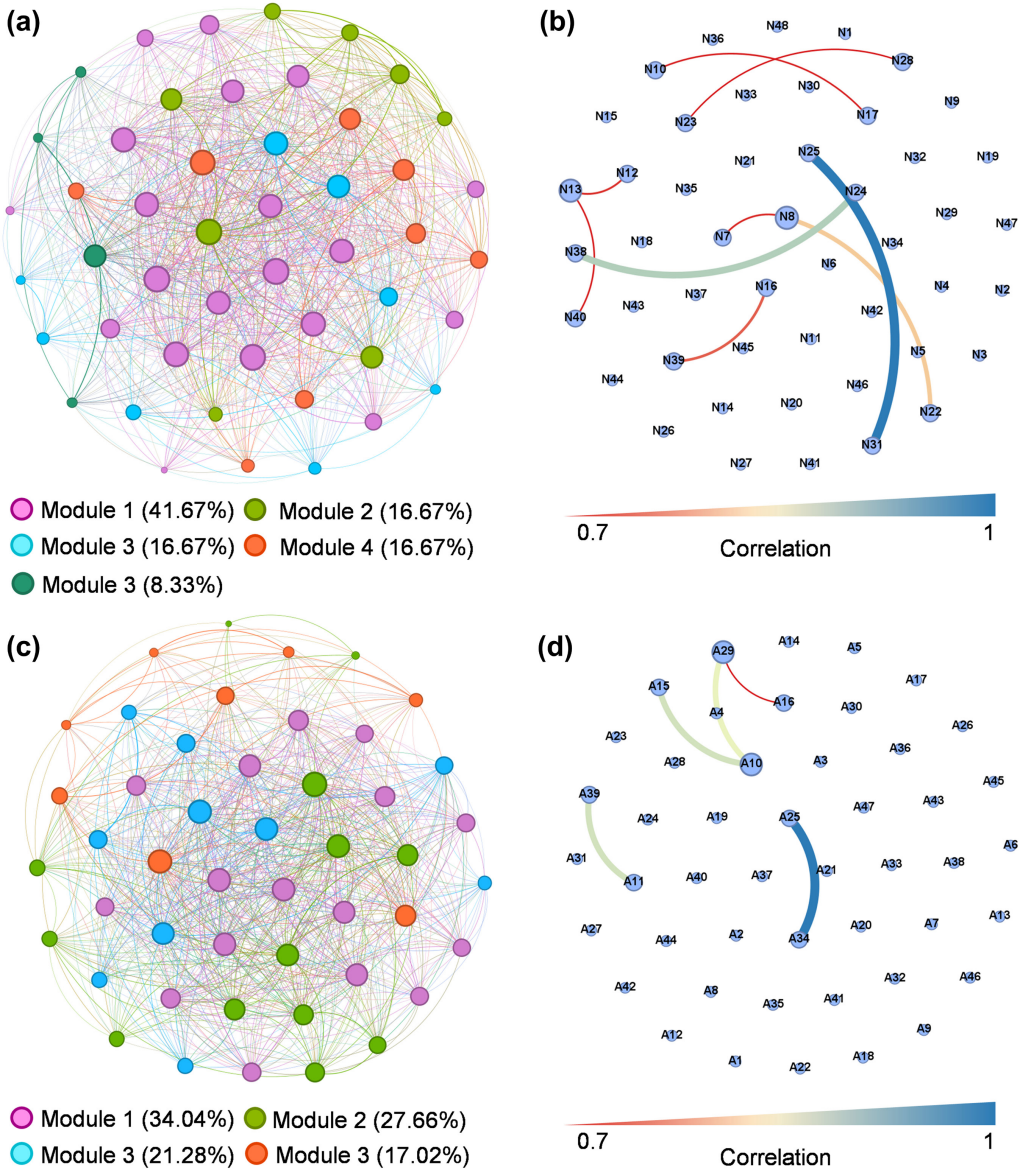
The co‐occurrence pattern of macrobenthic communities (a) and positive correlation of core species (b) at natural restoration sites. The co‐occurrence pattern of macrobenthic communities (c) and positive correlation of core species (d) at artificial restoration sites.

The network characteristics varied between the two groups, as shown in Figure 3. The biotic interaction network at natural restoration sites presented a slightly higher clustering coefficient (0.89 vs. 0.83) and lower modularity coefficient (0.08 vs. 0.11) than at artificial restoration sites, indicating higher complexity of the network and stronger interactions among macrobenthos communities at natural restoration sites. The macrobenthos communities at natural restoration sites demonstrated higher connecting lines (896 vs. 776) and average connection degree (37.3 vs. 33.0) but slightly lower average path length (1.21 vs. 1.28) than at artificial restoration sites.

**FIGURE 4:**
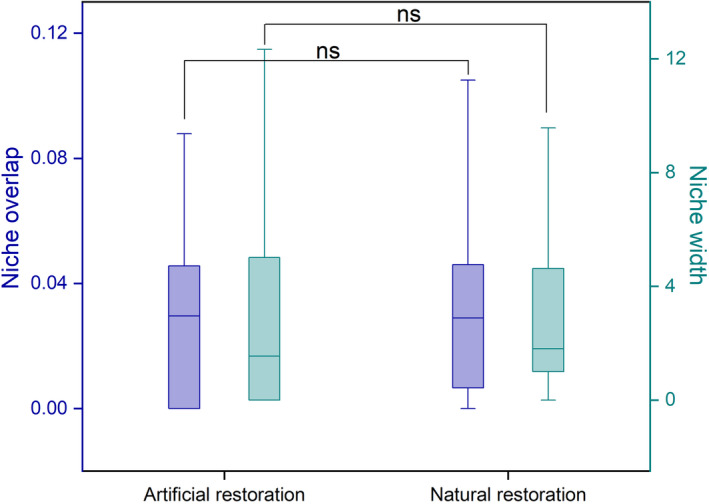
Observed niche overlap and niche width among species pairs within the macrobenthos at artificial and natural restoration sites. The median and quantile values from 25% to 75% were used in the boxplot. ns, *p* > .05.

Additionally, the macrobenthos network at the natural restoration sites showed higher ratios of positive correlations. The core macrobenthos species in the co‐occurring macrobenthos network of the artificial restoration sites were *Deiratonotus cristatum*, *Paracleistostoma cristatum*, *Sarmatium germaini* and *Taiwanassiminea hayasii* (Figure 3d). The core macrobenthic species in the co‐occurrence network of natural restoration sites were *Paracleistostoma cristatum*, *Deiratonotus cristatum*, *Paracleistostoma depressum*, *Paracleistostoma tomentosum*, *Cerithideopsis largillierti* and *Neripteron violaceum* (Figure 3b). A similar niche overlap and width among macrobenthos were also observed at natural and artificial restoration sites (Figure 4).

### 3.3 Macrobenthos community assembly in the artificial and natural restoration was both determined by deterministic processes

**FIGURE 5:**
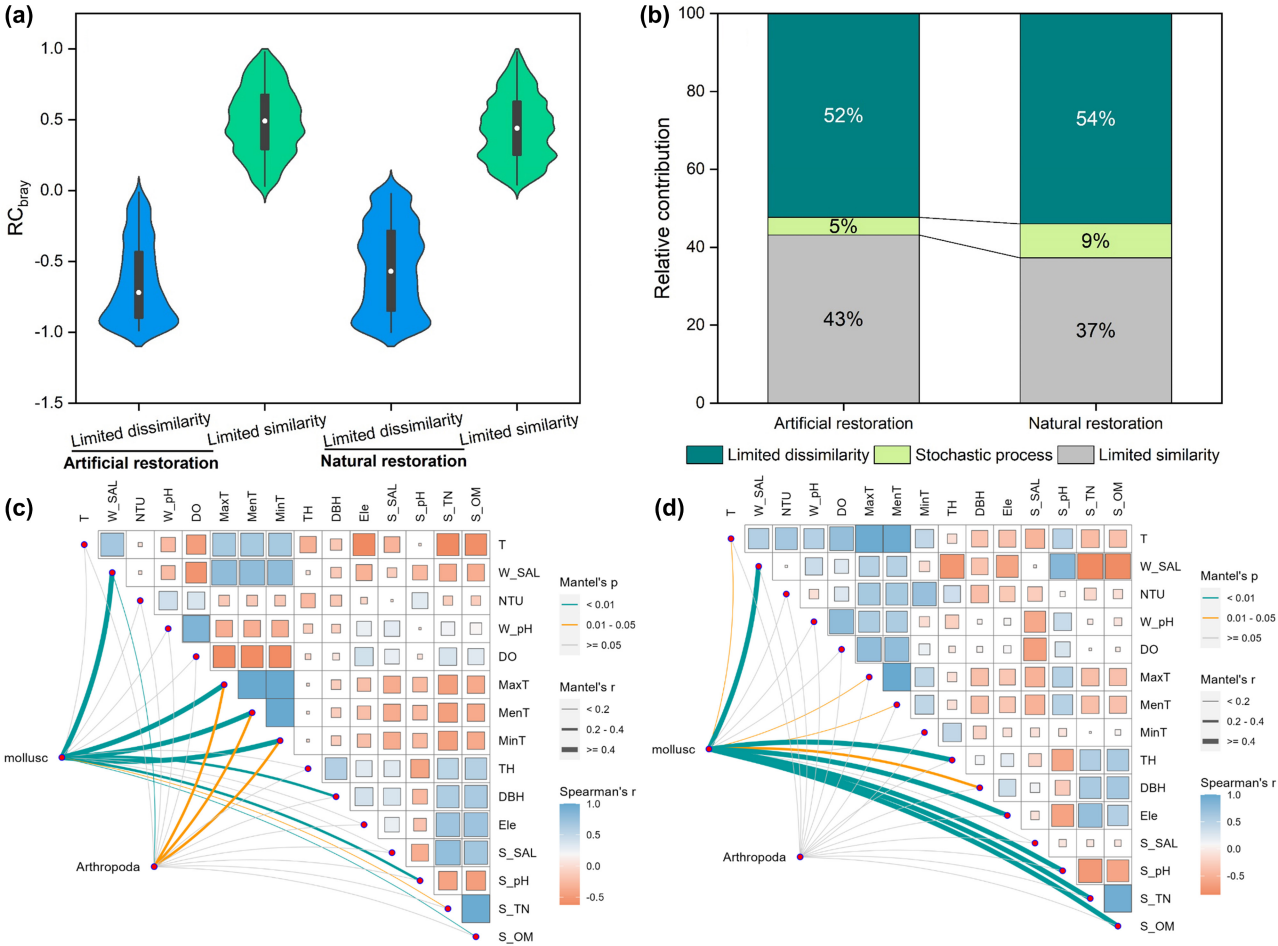
RCbray values (a) and relative importance of limiting similarity, dissimilarity and stochastic processes (b) in shaping the mangrove macrobenthos at artificial and natural restoration sites. The median and quantile values from 25% to 75% were used in the boxplot. Relationships between community dissimilarities and environment at natural restoration sites (c) and artificial restoration sites (d) based on the Mantel test and Spearman's rank correlation. Pairwise comparisons of the environment are shown in the upper right panel, with a colour gradient representing Spearman's correlation coefficients. Macrobenthos composition was correlated with each environment using Mantel tests. The line width represents Mantel's *r* statistic for the corresponding correlation, and the line colour indicates that the significance was tested based on 999 permutations. DBH, diameter at breast height; DO, seawater dissolved oxygen; Ele, elevation; MaxT, MenT; MinT, monthly maximum temperature, monthly average temperature, monthly minimum temperature; NTU, seawater turbidity; S_OM, soil total organic matter; S_pH, soil pH; S_SAL, soil salinity; S_TN, soil total nitrogen; T, seawater temperature; TH, tree height; W_pH, interstitial pH; W_SAL, interstitial salinity.

The β_*RC*_ results for the macrobenthos at the artificial restoration sites indicated that 43% had limited similarity, 52% had limited dissimilarity, and a few communities were spatiotemporally random (5%; Figure 5a,b). The β_*RC*_ results for macrobenthos at the natural restoration sites indicated that 37% had limited similarity, whereas 54% showed limited dissimilarity; a few communities were spatiotemporally random (9%; Figure 5a,b). Macrobenthic ecological processes at artificial restoration sites were jointly governed by community similarity and limiting community similarity (limiting similarity). However, the assembly of macrobenthic communities at natural restoration sites was dominated by environmental filtering (limiting dissimilarity).

The assembly of macrobenthos communities at the natural restoration sites was mainly driven by temperature and water resource competition (Figure 5c) but by soil and topography environment filtering and resource competition at the artificial restoration sites (Figure 5d). The relative abundances of molluscs and crustaceas at the natural restoration sites were significantly influenced by interstitial salinity (Mantel's *r* = .78, *p* = .003) and air temperature (Mantel's *r* = .69, *p* = .008), whereas the relative abundance of molluscs was also affected by soil pH (Mantel's *r* = .32, *p* = .009) and soil TOM (Mantel's *r* = .18, *p* = .006). The relative abundance of molluscs was mainly influenced by interstitial salinity (Mantel's *r* = .55, *p* = .002), elevation (Mantel's *r* = .52, *p* = .005), soil TN (Mantel's *r* = .75, *p* < .001), soil pH (Mantel's *r* = .81, *p* < .001), and soil TOM (Mantel's *r* = .80, *p* < .001) at the artificially restored sites. Interstitial salinity and soil physicochemical properties were the main factors affecting the relative abundance of mangrove macrobenthos at both artificial and natural restoration sites. Plant diameter at breast height and temperature had a large impact on macrobenthos at natural restoration sites.

## 4 DISCUSSION

There is a lack of effective evaluations from an animal diversity perspective regarding the efficacy of different abandoned pond‐to‐mangrove restoration methods. This evaluation is crucial for understanding the extent of macrobenthos recolonisation in mangrove restoration (Zhang et al., 2021). Furthermore, it provides significant guidance for managing and conserving regional and global biodiversity.

### 4.1 Human intervention did not provide more benefits in macrobenthic diversity during the process of abandoned pond‐to‐mangrove restoration

Some ecological restoration research proposed that artificial restoration is better than natural regeneration for the recovery of rainforest birds and plant diversity (Cardoso et al., 2022; Hariharan & Raman, 2022), and multi‐species mixed planting can considerably provide greater biodiversity and ecosystem function in the early stages of restoration (Feng et al., 2022). At the same time, some recent studies agree that natural regeneration produce greater benefits in enhancing biodiversity and restoring ecosystem functions than artificial restoration (Crouzeilles et al., 2017; Hua et al., 2022). However, these evidence mainly derived from studies on the restoration of terrestrial ecosystems, little is known about which restoration strategy (natural or artificial restoration) provides more benefits to the biodiversity of coastal habitats, especially to macrobenthic diversity. Therefore, our study aims to address this knowledge gap. We found that the observed species richness and the α‐diversity indices of mangrove macrobenthos at the artificial restoration sites were similar to or slightly lower than those at the natural restoration sites. The reason is that artificially restored ecosystems often lack the full range of functional traits found in natural regeneration sites, and natural regeneration can restore the natural balance of ecosystems due to natural recruitment and environmental conditions, thus may supporting higher biodiversity (Crouzeilles et al., 2017), as well as macrobenthos diversity. Human intervention has led not only to the lower diversity of mangrove macrobenthos but also to a reduction in variation of animal community composition due to mangrove plants homogenisation. We found that the Bray–Curtis dissimilarity index of macrobenthos at the artificial restoration sites was significantly lower than that at the natural restoration sites. Large‐scale introduction and planting enhanced the local diversity of mangroves but increased biological homogenisation. Biological homogenisation affects mangrove functionality (Chen, Gu, Lee, et al., 2021), further declining ecosystem stability and increasing vulnerability (Wang et al., 2021). For example, human intervention involving the ecological domestication of plants (e.g. cold and/or high salinity adaptation) could disrupt the eco‐physiological barriers of mangroves (Larson et al., 2014). Fifteen mangrove species have expanded to higher latitudes due to human intervention in China, breaking through natural barriers (Hickling et al., 2006; McGill et al., 2015; Tom et al., 2019). Two exotic plants, *Sonneratia apetala* and *Laguncularia racemosa*, which cover an area of more than 3800 ha in China, have led to adverse ecological invasions (He et al., 2018; Lee et al., 2019; Wang et al., 2021). The introduction of exotic species will lead to a reduction in the ecological niches of some native species, more intense competition for limited resources and a reduction in the distribution of rare and endangered species or even their disappearance from the region (Chen, Gu, Liu, et al., 2021; Zhang et al., 2021). Therefore, in addition to focusing on the restoration area and survival rate of mangrove forests, the overall functional restoration of mangrove wetland ecosystems needs to be paid more attention. We should incorporate animal diversity, disaster prevention and mitigation capacity, and carbon sequestration into the restoration objectives to improve the quality of mangrove ecosystems in future pond mangrove restoration projects.

### 4.2 Natural‐based solution could shape more complex community structures and higher biotic heterogeneity than artificial restoration method

In the past, many restoration projects (e.g. tidal flat afforestation and direct seedling insertion) have ignored the tolerance of mangroves to extreme environmental factors, such as inundation, water salinity and soil salinity, which results in low effectiveness of mangrove restoration (Lee et al., 2019; Wang et al., 2021). The afforestation of mangrove forests on tidal flats in China (e.g. Guangxi Province) had an effectiveness rate of only 37.1% between 2002 and 2007, which decreased to 26.6% between 2008 and 2015, resulting in a 10.5% decrease in effectiveness (Fan & Mo, 2018). Artificially planted mangroves in unsuitable habitats result in low seedling survival rates, as also observed in Bangladesh, the Philippines and Sri Lanka (Lewis, 2005; Wang et al., 2021). Due to limited accommodation space for tidal flats afforestation and large‐scale planting‐induced resource waste worldwide (Lee et al., 2019; Lovelock & Brown, 2019), abandoned pond‐to‐mangrove restoration should be the primary method for future mangrove restoration worldwide. The total area of abandoned aquaculture ponds in China is approximately 73,000 ha (Friess et al., 2019; Wang & Wang, 2007), of which the area of aquaculture ponds in mangrove reserves and wetland parks is nearly 10,000 ha. Most of these ponds were formerly mangrove forests. A large area of fishponds within the reserve urgently needs pond retirement and forest return. The Administration of Hainan Dongzhaigang National Nature Reserve implemented pond retirement and forest returns for 140 ha of fishponds in the reserve in 2014. Since then, several ponds have been returned to forests in the southern provinces and regions of China. Several studies have suggested that mangroves cannot recover naturally after aquaculture ponds are abandoned because of the blocked hydrological flow (Matsui et al., 2010). From a technical standpoint, the key to the success or failure of mangrove ecological restoration is to determine the hydrological conditions (mudflat elevation, inundation length, inundation frequency and inundation depth) of the existing mangrove forests in and around the restoration area (Lewis, 2005; Wu et al., 2021). Opening the embankment gap to create a waterway channel is a major action at abandoned pond‐to‐mangrove restoration sites (Zhao et al., 2016). Converting ponds to forests requires restoration of the connectivity of the ponds to the outer sea hydrography. An adequate supply of propagules is a prerequisite for natural restoration, and artificial planting is the last choice (Di Nitto et al., 2014; Lewis, 2005). We recommend focusing on natural restoration methods and appropriately increasing artificial intervention in abandoned ponds for future mangrove restoration projects. The natural recovery of mangrove forests can be completed within 15–30 years if hydrological conditions are restored (Kamali & Hashim, 2011; Song et al., 2023). Abandoned ponds in Thammarat Province, Thailand, were restored to hydrological connectivity, and propagules of 15 mangrove plant species naturally drifted in and colonised after 6 years, with artificial plantings of *Kandelia obovata* and *Rhizophora apiculata* at the same heights as those that drifted in (Matsui et al., 2010). Compared with artificial restoration, natural restoration has the advantages of low input and good community structure (Fan & Mo, 2018; Wang et al., 2021). Our results showed that the Shannon–Wiener, Simpson, Pielou and Bray–Curtis phase‐difference indices of macrobenthos at artificial restoration sites were smaller than those at natural restoration sites. Macrobenthos species at artificial restoration sites were more competitive than those at natural restoration sites. We also found stronger connectivity, more pronounced interactions and higher network complexity among macrobenthos at the natural restoration sites than at the artificial restoration sites. Habitat heterogeneity and multiple‐species planting were important for macrobenthic community resistance to environmental changes (Leung, 2015). The stability of mangrove macrobenthos at artificial restoration sites is poor because single‐species was planted. Although many scholars have recognised the importance of natural restoration of mangrove forests (Fan & Mo, 2018; Wang et al., 2021; Zhang et al., 2021), existing scientific research assessment systems, funding systems and performance assessment systems are not conducive to natural restoration, and the effectiveness of natural restoration of China's mangrove forests has not been positively evaluated. This has reduced the space available for natural restoration significantly.

### 4.3 Deterministic processes dominate the macrobenthos community assembly by controlling soil and interstitial factors in both natural and artificial restoration

Illustrating macrobenthos community assembly mechanisms can improve the understanding of enhancing the functions and services of restored mangrove forests. Deterministic processes include selection based on abiotic environmental factors and interactions between species. Stochastic processes include unpredictable disturbances, probabilistic diffusion and random birth‐death events (Stegen et al., 2012; Zhao et al., 2022). Deterministic and stochastic processes are essential to reveal the mechanisms of species coexistence and biodiversity maintenance in mangrove animal communities (Chen, Gu, Lee, et al., 2021). Our results suggest that macrobenthos community assembly was primarily influenced by deterministic processes in both natural and artificial restoration sites, such as environmental filtering and competition. Environmental filtering explained 52% and 54% of the variation in mangrove macrobenthic structure at the artificial and natural restoration sites, respectively. This indicated that deterministic processes are more important than stochastic processes in shaping the macrobenthos communities under both artificial and natural restoration. Artificial restoration sites had severe human interference, which made the macrobenthos communities sensitive to environmental changes induced by human activities (Chen et al., 2023). Although there is less human disturbance in natural restoration sites, changed tidal and soil elements can impact the species composition and competition pattern of macrobenthic communities (Zhang et al., 2022), which determines the macrobenthic community assembly. Soil physicochemical properties and interstitial salinity influence mangrove macrobenthos distribution. Artificial soil tillage in the pond changed soil properties, resulting in a greater influence of soil on mangrove macrobenthos at artificial restoration sites than at natural restoration sites. Research has demonstrated that habitat heterogeneity in mangrove ecosystems is very high, with temperature, seawater salinity, pH, tidal level and mangrove type affecting mangrove macrobenthos (Wu, 2016). At the artificial and natural restoration sites, species competition explained 43% and 37% of the variation in mangrove macrobenthic structure, respectively. Macrobenthos have strong dispersal ability under the action of currents and tides and can spread and colonise large spatial scales, such as estuaries and intertidal zones, thus generating species competition (D'Aloia et al., 2022; Leibold et al., 2004).

## 5 CONCLUSIONS

The conservation and restoration of mangrove forests in China have implications for global mangrove conservation and restoration. China is one of the few countries showing a net increase in mangrove areas. The limited accommodation space for tidal flat afforestation and large‐scale planting has resulted in resource wastage. At the same time, a large of abandoned ponds within the natural reserve are urgently needed to be restored for mangrove forests. As a better solution than tidal flat reforestation, abandoned ponds are advantageous for restoring mangrove ecosystem functions. This will be an important method for mangrove restoration in the future. We found that artificial restoration did not provide more benefits to macrobenthic biodiversity than natural restoration. Furthermore, natural restoration as a nature‐based solution could produce higher more complex community structure and higher species interaction than artificial restoration. Although there was stronger connectivity and interactions among the macrobenthos species at the natural restoration sites, natural regeneration is sluggish and slow efficient, with uncertainties such as the slow pace of natural population dispersal and the sensitivity of the biological environment. Therefore, natural restoration, as the main method with artificial restoration as a supplement, is recommended for future mangrove restoration. Deterministic processes should be primarily considered when designing management programmes to restore and conserve mangrove biodiversity. An operational manual for abandoned pond‐to‐mangrove restoration should be developed to provide a basis for implementing abandoned ponds for mangrove restoration in China to improve mangrove conservation. In addition, more effort should be focused on identifying hotspots and priorities for mangrove restoration.

## AUTHOR CONTRIBUTIONS

**Pingping Guo:** Conceptualization (lead); data curation (lead); formal analysis (lead); methodology (lead); visualization (lead); writing – original draft (lead); writing – review and editing (lead). **Yufeng Lin:** Data curation (equal); investigation (equal); writing – review and editing (equal). **Yifei Sheng:** Data curation (equal); investigation (equal). **Xuan Gu:** Data curation (equal). **Yijuan Deng:** Data curation (equal). **Yamian Zhang:** Data curation (equal). **Wenqing Wang:** Funding acquisition (equal); writing – review and editing (equal). **Mao Wang:** Conceptualization (equal); funding acquisition (lead); supervision (lead); writing – review and editing (equal).

## FUNDING INFORMATION

This study was funded by the National Natural Science Foundation of China, Grant/Award Number: 42076161 and 42176169.

## CONFLICT OF INTEREST STATEMENT

The authors declare no conflict of interest.

## Supporting information


Appendix S1

## Data Availability

All associated raw data to reproduce the results presented in this article are available on Figshare (https://doi.org/10.6084/m9.figshare.25971811).
