# Supplementary material for: Comparison of the coexistence pattern of mangrove macrobenthos between natural and artificial reforestation
Source: Ecol Evol. 2024 Jul 31;14(8):e70069. doi: 10.1002/ece3.70069 (PMC11289789; doi:10.1002/ece3.70069)
Supplement: Supplementary file 1 — Appendix S1 [file ECE3-14-e70069-s001.docx]

**Supplementary Information for**

**Comparison of the coexistence pattern of mangrove macrobenthos between natural** **and artificial reforestation**

Pingping Guo^1, 2^︱Yufeng Lin^1, 2^︱Yifei Sheng^1, 2^︱Xuan Gu^1, 2^︱Yijuan Deng^1, 2^︱Yamian Zhang^1, 2^︱Wenqing Wang^1, 2^︱Mao Wang^1, 2^

^1^ Key Laboratory of the Ministry of Education for Coastal and Wetland Ecosystems, College of the Environment & Ecology, Xiamen University, Xiamen, China; ^2^ Zhangjiang Estuary Mangrove Wetland Ecosystem Station, National Observation and Research Station for the Taiwan Strait Marine Ecosystem, Xiamen University, zhangzhou China

Correspondence

Mao Wang

Email: [wangmao@xmu.edu.cn](mailto:wangmao@xmu.edu.cn)

**This file includes:**

TABLES S1-2

FIGURES S1

TABLE S1 The code for each species in natural restoration (NR) and artificial restoration (AR), Dongzhaigang Bay (figures 3).

| Species | Code |
| --- | --- |
| *Cerithidea ornata* | N1/A1 |
| *Pirenella incisa* | N2/A2 |
| *Littoraria scabra* | A3 |
| *Optediceros breviculum* | N3/A4 |
| *Batillaria multiformis* | A5 |
| *lravadia quadrasi* | N4 |
| *Pseudomphala latericea* | N5/A6 |
| *Parasesarma maipoense* | N6/A7 |
| *Geloina coaxans* | N7/A8 |
| *Cerithideopsis largillierti* | N8/A9 |
| *Laternula truncata* | N9 |
| *Turritella bacillum* | N10 |
| *Taiwanassiminea hayasii* | A10 |
| *Onchidium struma* | N11/A11 |
| *Melanoides tuberculata* | A12 |
| *Ellobium chinense* | N12 |
| *lravadia* sp.1 | N13/A13 |
| *lravadia* sp.2 | A14 |
| *Neritina paralella* | A15 |
| *Stenothyra japonica* | N14/A16 |
| *Pythia trigona* | A17 |
| *Salinator sanchezi* | N15 |
| *Onchidium verruculatum* | A18 |
| *Indoaustriella scarlatoi* | N16/A19 |
| *Cycladicama ethima* | N17 |
| *Pirenella microptera* | N18/A20 |
| *Sermyla riqueti* | N19 |
| *Indoaustriella plicifera* | N20 |
| *Pirenella cingulata* | N21/A21 |
| *Neripteron violaceum* | N22/A22 |
| *Uca borealis* | N23/A23 |
| *Paracleistostoma depressum* | N24/A24 |
| *Deiratonotus cristatum* | N25/A25 |
| *Neosarmatium smithi* | A26 |
| *Thalamita crenata* | N26/A27 |
| *Uca arcuata* | N27/A28 |
| *Sarmatium germaini* | N28/A29 |
| *Sesarma plicata* | N29/A30 |
| *Ilyoplax serrata* | A31 |
| *Scylla serrata* | A32 |
| *Parasesarma eumolpe* | N30/A33 |
| *Paracleistostoma cristatum* | N31/A34 |
| *Terebralia sulcata* | N32 |
| *Episesarma mederi* | A35 |
| *Macrophthalmus definitus* | N33/A36 |
| *Uca paradussumieri* | N34/A37 |
| *Scylla paramamosain* | N35/A38 |
| *Episesarma versicolor* | N36/A39 |
| *Uca lactea* | A40 |
| *Macrophthalmus tomentosus* | N37/A41 |
| *Paracleistostoma tomentosum* | N38/A42 |
| *Perisesarma bidens* | N39/A43 |
| *Scopimera curtelsoma* | N40 |
| *Tubuca typhoni* | N41 |
| *Macrophthalmus pacificus* | N42 |
| *Macrophthalmus convexus* | N43 |
| *Metaplax elegans* | N44/A44 |
| *Scopimera globosa* | N45/A45 |
| *Metaplax longipes* | N46/A46 |
| *Varuna litterata* | N47/A47 |
| *Macrophthalmus erato* | N48 |

TABLE S2 Percentage contribution (%) of discriminating mangrove macrobenthos to the average dissimilarity between natural restoration (NR) and artificial restoration (AR), in Dongzhaigang Bay.

| Mangrove macrobenthos | Contribution (%) | |
| --- | --- | --- |
| *Pirenella microptera* | 20.06 |  |
| *Pirenella cingulata* | 7.03 |  |
| *Neripteron violaceum* | 5.68 |  |
| *Pirenella incisa* | 5.34 |  |
| *Cerithideopsis largillierti* | 4.49 |  |
| *Optediceros breviculum* | 4.06 |  |
| Cumulative contribution | 46.66 | |


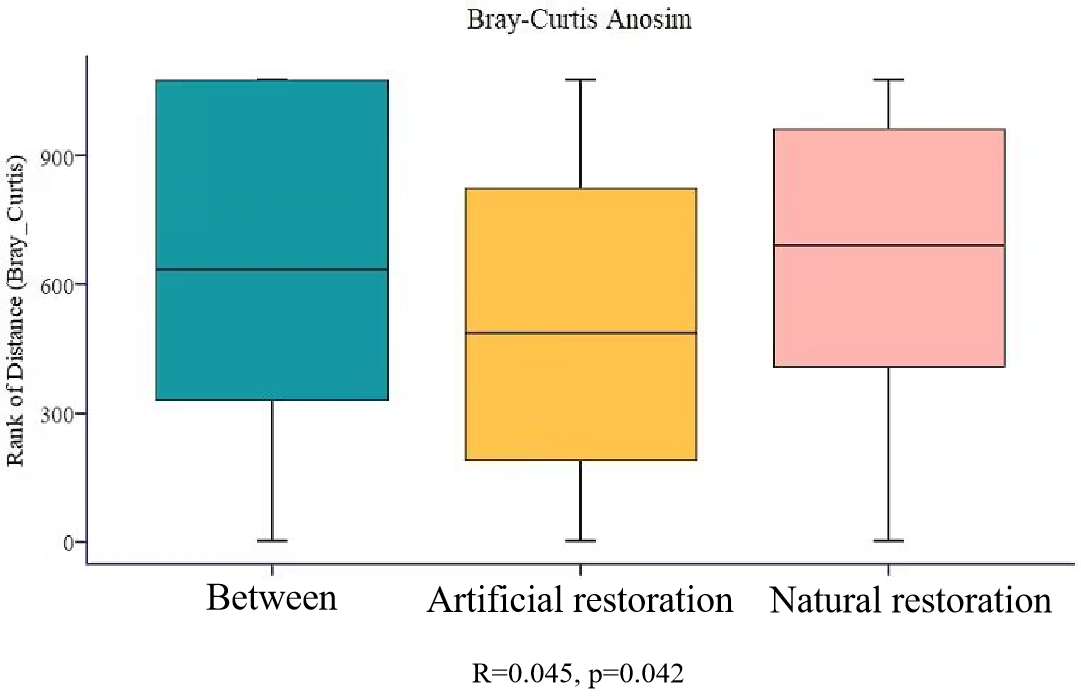


FIGURE S1. Ranking of distances among mangrove macrobenthos between natural and artificial restoration.
